# Supplementary material for: Health Behaviors and Self-Reported Oral Health among Centenarians in Nanjing, China: A Cross-Sectional Study
Source: Int J Environ Res Public Health. 2021 Jul 7;18(14):7285. doi: 10.3390/ijerph18147285 (PMC8304003; doi:10.3390/ijerph18147285)
Supplement: Supplementary file 1 [file ijerph-18-07285-s001.zip › ijerph-1260465-supplementary.pdf]

International Journal of
Environmental Research and Public Health

MDPI

Table S1. Measurement of oral health variables in Nanjing Centenarians Study (n = 185).

Variables	Survey questions	Measurement
**Oral health outcomes**		
Self-reported oral health	What is the condition of your mouth and teeth?	1 = very poor, 2 = poor, 3 = fair, 4 = good, and 5 = very good
Edentulous status	Number of natural teeth?	0 = lost all-natural teeth and 1 = dentate (having one or more natural teeth)
**Health behaviors**		
Former/current smoker	Did you smoke in the past/present?	0 = non-smoker, 1 = former or current smoker
Eating fruits every day	How often have you eaten fruits in the last two years?	1 = never, 2 = sometimes, 3 = at least once a month, 4 = at least once a week, and 5 = every day
Eating vegetables every day	How often have you eaten vegetables in the last two years?	1 = never, 2 = sometimes, 3 = at least once a month, 4 = at least once a week, and 5 = every day
Leisure activities	How often do you participate in the following leisure activities?	0 = no participation, 1 = sometimes/at least once a month/at least once a week/every day
Oral hygiene behavior	How often do you brush your teeth (dentures or gums)?	0 = never, 1 = occasionally, 2 = once a day, 3 = twice a day or more
**Confounders**		
Age	How old are you?	Continuous variable
Gender	Your gender?	0 = woman, 1 = man
Education	Years of schooling?	0 = having no formal education, 1 = having formal education
Annual household income	Income per capita of your household last year?	Continuous variable
Co-residence	Co-residence of interviewee?	0 = not living with family members, 1 = living with family members
ADL dependent	Were you limited in activities (bathing, dressing, toileting, indoor transferring, and continence, eating) because of health problem?	1 = without assistance, 2 = one part assistance, 3 = more than one part assistance
Cognitive function	Short validated Chinese version of the Mini-Mental State Examination (MMSE) *	Continuous variable
Chronic conditions	Suffering from hypertension, diabetes mellitus, heart disease, stroke, lung disease, cancer, and arthritis?	0 = no, 1 = yes

* MMSE included the following questions:

(1) What time of day is it right now?
(2) What is the animal year of this year?
(3) What is the date of spring festival?
(4) What is the season right now?
(5) What is the name of this county or district?

(6) Of kinds of food named in one minute.
(7) Repeat the name of "table" at the first time.
(8) Repeat the name of "apple" at the first time.
(9) Repeat the name of "clothes" at the first time.
(10) RMB20-RMB3=?
(11) RMB20-RMB3-RMB3=?
(12) RMB20-RMB3-RMB3-RMB3=?
(13) RMB20-RMB3-RMB3-RMB3-RMB3=?
(14) RMB20-RMB3-RMB3-RMB3-RMB3-RMB3=?
(15) Repeat the name of "table" a while later.
(16) Repeat the name of "apple" a while later.
(17) Repeat the name of "clothes" a while later.
(18) Naming "pen".
(19) Naming "watch".
(20) Repeat a sentence.
(21) Taking paper using right hand.
(22) Folding paper.
(23) Put paper on the floor.

Options: 0 = wrong, 1 = correct, 8 = not able to do/answer, 9 = missing
